# Total hip arthroplasty via the direct anterior approach with Kerboull-type acetabular reinforcement device for an elderly female with factor XI deficiency

**DOI:** 10.1051/sicotj/2016046

**Published:** 2017-02-13

**Authors:** Kei Sano, Yasuhiro Homma, Tomonori Baba, Jun Ando, Mikio Matsumoto, Hideo Kobayashi, Takahito Yuasa, Kazuo Kaneko

**Affiliations:** 1 Department of Orthopaedic Surgery, Juntendo University Tokyo 113-0033 Japan; 2 Division of Hematology, Department of Internal Medicine, Juntendo University Tokyo 113-0033 Japan

**Keywords:** Total hip arthroplasty, Direct anterior approach, Factor XI deficiency, Kerboull-type acetabular reinforcement device

## Abstract

We present a case of successful and uncomplicated total hip arthroplasty with an acetabular reinforcement device in an elderly patient with hip osteoarthritis already diagnosed with factor XI deficiency, which is a very rare bleeding disorder and at high risk of post-operative haemorrhage, and it poses a substantial challenge to surgeons as a consequence of the specific risks of infection and fixation failure. Moreover, bone fragility in elderly patient increases potential risk of adverse event. Fresh frozen plasma was used to supplement factor XI activity. Importantly, transfusion-transmitted disease such as having factor XI inhibitor was promptly surveyed prior to the supplement since the patient had previous history of the administration of fresh frozen plasma. Under prompt and effective peri-operative haemostasis, rigid implant fixation and rigorous attention to the prevention of infection seem to achieve the best possible outcomes for elderly patients with a bleeding disorder undergoing total hip arthroplasty.

## Introduction

Factor XI (FXI) deficiency, also known as haemophilia C, an autosomal recessive bleeding disorder, was first described in the 1950s [1]. The estimated overall prevalence of severe FXI deficiency is 1:1 000 000 [2]. Affected patients are often asymptomatic until they undergo surgery or experience trauma. Consequently, the diagnosis is most frequently made in late childhood or early adulthood. Patients with severe FXI deficiency are at high risk of post-operative haemorrhage, in common with those with haemophilia A, haemophilia B or other coagulation factor deficiencies. Guidelines are available to inform the management of unexpected massive haemorrhage in those with known bleeding disorders, and the peri-operative management of those requiring surgery [3, 4]. Nonetheless, there have been few studies of arthroplasty in patients with FXI deficiency [5]. In addition to the general risks to the patient of peri-operative haemorrhage, major orthopaedic joint replacement surgery in patients with a bleeding disorder poses a substantial challenge to clinicians as a consequence of the specific risks of infection and fixation failure [6, 7]. Moreover, bone fragility in elderly patient increases potential risk of adverse event. Here, we present a case of successful and uncomplicated total hip arthroplasty (THA) with an acetabular reinforcement device in an elderly patient with hip osteoarthritis already diagnosed with FXI deficiency.

## Case report

**Figure 1.**
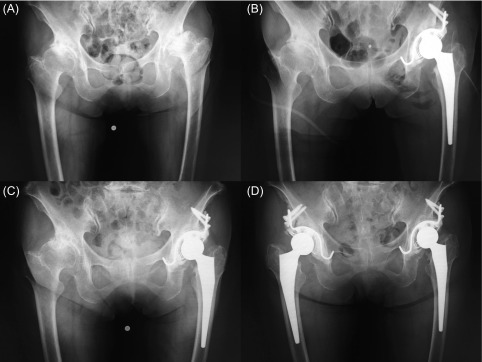
(A) Osteoarthritis of the bilateral hip, (B) immediate post-operative x-ray of the left hip, (C) 12 months after the operation. Right hip required THA, (D) there is no sign of implant loosening at 12 month after right THA, 24 months after left THA.

A 77-year-old woman presented to our hospital complaining of severe and debilitating left hip pain. A pelvic radiograph showed end-stage osteoarthritis of the bilateral hip (Figure 1A). She was found to have FXI deficiency in her 40s, as a result of family screening shortly after her brother had been diagnosed with the same complaint. At the age of 70 years, she had undergone laparotomy for partial colectomy, when her peri-operative management included the administration of fresh frozen plasma (FFP) and red cell concentrate (RCC). After a full explanation of the benefits and risks of THA, a written informed consent was obtained. She also gave consent for the publication of her clinical data.

Pre-operative investigations revealed normal renal and hepatic function. The prothrombin time and international normalised ratio were both normal at 13.2 s and 1.02, respectively. Activated partial thromboplastin time (APTT) was substantially prolonged at 96.2 s. The platelet count was normal. The plasma activity of FXI was <1%, confirmed the diagnosis of FXI deficiency. The activities of factors VIII and IX were normal (147% and 87.1%, respectively). As the patient had previously been the recipient of FFP, cross-mixed screening for anti-FXI antibodies was undertaken and proved to be negative.

Four units of FFP were transfused the day before surgery, and a further four units on the day of the operation (total eight units). After transfusion, the APTT had improved to 45.6 s. Pre-operative serum haemoglobin concentration (Hb) was 12.9 g/dL and haematocrit 39.6%. We elected not to collect the patient’s blood after the induction of general anaesthesia for later autologous transfusion due to the risk of haemorrhage during collection. Instead, we kept four units of RCC and two units of FFP on standby during the operation, and intra-operative blood salvage was performed.

Intravenous ampicillin/sulbactam 3.0 g was administered after induction of anaesthesia, 15 min before the skin incision was made. The direct anterior approach to THA was undertaken using a Kerboull-type acetabular reinforcement device with X3 RimFit (Stryker Orthopaedics, Mahwah, NJ, USA) secured in antibiotic-loaded acryl cement (1 g vancomycin per 40 g of cement; Surgical Simplex^®^, Stryker Orthopaedics) (Figure 1B). A non-cemented proximally coated tapering stem (Accolade TMZF^®^, Stryker Orthopaedics) was inserted. The operative time was 129 min; blood loss was 840 mL. Only blood salvaged intra-operatively was administered during surgery. Immediately after surgery, Hb was 10.3 g/dL and haematocrit 30.1%. On the first post-operative day, a surgical drain was removed in which 159 mL had accumulated overnight. Routine post-operative laboratory tests found an APTT of 43.4 s and Hb of 9.0 g/dL; consequently two units of RCC were transfused. Two further 1.5-g doses of ampicillin/sulbactam were administered on the first post-operative day. On the third post-operative day, APTT was 63.8 s and Hb was 9.9 g/dL; no further FFP or RCC was transfused. Full weight bearing was allowed immediately after surgery. Wound healing was uncomplicated, and the rest of her recovery was uneventful. The patient was discharged home on the 32th post-operative day.

One month after surgery, APTT was 85.1 s and Hb was 12.4 g/dL. During the follow-up periods, right THA was performed due to a development of severe right hip pain (Figure 1C). Exactly the same peri-operative management and the operative protocol were performed, and similar post-operative course without complication was observed. At 12 months for right hip and 24 months for left hip after the operation, there were no signs of bleeding, infection or implant loosening (Figure 1D). The Harris Hip Score improved from 37 pre-operatively (initial operation) to 93.4 post-operatively.

## Discussion

Total hip arthroplasty is a highly effective orthopaedic intervention. Advances in peri-operative medical care, and improved operative skills and materials [8, 9], have resulted in a very low incidence of complications during and after THA [10]. Nevertheless, patients with rare and potentially serious comorbid disease require more intensive peri-operative management to avoid adverse events.

Factor XI deficiency is a very rare bleeding disorder. Unlike patients with other inherited coagulation factor deficiencies such as haemophilia A or B, patients with FXI deficiency rarely bleed spontaneously. Ragni et al*.* reported that in 25 related FXI-deficient individuals, none experienced deep muscle haematoma, haemarthrosis or bleeding into the central nervous system, gastrointestinal tract or retroperitoneal space [11]. Massive bleeding has, however, been reported in selected cases of trauma or surgery [12, 13]. Dempfle et al*.* reported a patient with FXI deficiency who had severe post-operative bleeding after cholecystectomy, requiring massive transfusion [13]. Although rare, surgeons and anaesthesiologists should consider unrecognised bleeding disorders such as FXI deficiency if there is torrential unexpected and unexplained peri-operative bleeding. In those in whom FXI deficiency has already been diagnosed, a comprehensive management strategy should be identified and acted upon by all clinicians involved in the patient’s care.

We used FFP to supplement FXI activity in this case. The volume required depends on the severity of the deficiency and the extent of surgery or trauma. Yamada et al. reported transfusing ten units of FFP during the successful peri-operative management of a patient with FXI deficiency and femoral neck fracture undergoing bipolar hip arthroplasty [14], broadly comparable with the eight units that we used for a THA. The target FXI activity for haemostasis is reportedly 15–30% [15], and peri-operative monitoring is considered desirable, but it is often not practical due to the time needed to process assays and greater costs. It has also been reported that FXI activity might not correlate well with bleeding tendency [16]. We elected instead to monitor APTT, in accordance with other reports [14]. In our case and that of Yamada et al., APTT remained elevated at 45.6 s and 52.0 s, respectively. Given the positive outcomes, it appears that maintaining APTT in the range 40–50 s is sufficient for haemostasis [14, 17]. The transfusion of more than ten units of FFP may not be appropriate in patients at the extremes of age or with cardiovascular compromise, due to the risk of intravascular volume overload; the risks of transfusion reaction and transfusion-transmitted disease must also be taken into account [3, 12]. Salomon et al*.* reported that seven of 21 patients with FXI deficiency (33.3%) who had received FFP were subsequently found to have developed FXI inhibitor [12]. Thus FXI inhibitor should be examined if the patient had a history of previous supplementation, otherwise the patient might develop mild to severe allergic reaction [12].

Factor XI concentrate is available as an alternative to FFP in some countries, but not in Japan. Factor XI concentrate is virally inactivated and can be used to achieve FXI plasma activity sufficient for haemostasis with short infusion times, without volume overload and with a lower risk of allergic reaction [5]. Santoro et al*.* have reported the uncomplicated conduct of hip arthroplasty in a patient with FXI deficiency who had previously experienced a severe allergic reaction to plasma using Hemoleven^®^ FXI concentrate (LFB Biomedicaments, Les Ulis, France) [5]. Desmopressin, recombinant activated factor VII and tranexamic acid are also reported to be effective for the management of FXI deficiency [3, 13, 18]. We chose FFP as the first-line treatment for our patient as there was no evidence to suggest that she was at risk of fluid overload or allergic reaction.

The outcomes after THA in patients with haemophilia are reportedly less favourable than those without [6, 7]. Although the peri-operative management of haemostasis has improved markedly in recent years, specific implant-related complications such as loosening and infections must still be addressed. Furthermore, unlike haemophilia A and B, patients with factor XI deficiency are relatively much older at the time of joint replacement surgery, where additional risk “bone fragility” should be considered. We chose the Kerboull-type reinforced device in order to ensure rigid biomechanical fixation was achieved [19, 20]. Although this device required greater exposure around the acetabular site, we successfully implanted it without excessive bleeding using a minimally invasive intermuscular and internervous anterior approach that does not require muscle transection to reach the hip joint [21]. The reasons why we used those devices were that judging from the recent literature (Table 1), better clinical outcomes appear to be achieved with modern non-cemented implants compared with traditional cemented implants [6, 7, 22–24]; however, the incidence of loosening of non-cemented cups is still high in patients with bleeding disorder, even in younger study populations [6, 7, 22]. Moreover, our patient was 77 years old, her bone quality did not seem adequate for press-fit fixation. We also used an antibiotic-loaded acryl cement to reduce the risk of infection [25]. We used a non-cemented stem, which is our routine clinical practice and chimes with a recent report that there was no long-term evidence of loosening of a non-cemented stem in patients who underwent cementless THA for haemophilic arthropathy [22].

**Table 1.** Previous articles of THAs in patients with haemophilia.

Author	Year	Fixation	Number	Follow-up (years)	Deep infection rate (%)	Cup revision rate (%)	Stem revision rate (%)	Total re-operation rate (%)
Nelson et al.	1992	Cement	22	7.6	9.1	NA	27.2	36.4
Kelly et al.	1995	Cement	26	8	11.5	23	21	23
Miles et al.	2008	Cement	16	6.25	NA	NA	NA	18.8
Yoo et al.	2009	Non-cement	27	7.7	0	7.4	0	11.1
Lee et al.	2015	Non-cement	21	10.1	0	9.5*	0	14.3

NA: Not Available.

* Liner and head change included.

## Conclusion

Our peri-operative management strategy proved to be effective for major joint arthroplasty in a patient with FXI deficiency. Our case highlights the importance of prompt and effective peri-operative haemostasis, rigid implant fixation and rigorous attention to the prevention of infection in achieving the best possible outcomes for patients with a bleeding disorder undergoing THA.

## Conflict of interest

The authors declare no conflict of interest in relation with this paper.
